# In vitro gametogenesis: The end of egg donation?

**DOI:** 10.1111/bioe.12499

**Published:** 2018-08-23

**Authors:** Sarah Carter‐Walshaw

**Affiliations:** ^1^ Department of Politics, Philosophy and Religion Lancaster University Lancaster United Kingdom

**Keywords:** artificial gametes, egg donation, in vitro gametogenesis, IVG, reproductive donation

## Abstract

This paper explores whether egg donation could still be ethically justified if in vitro gametogenesis (IVG) became reliable and safe. In order to do this, issues and concerns that might inform a patient’s reasoning in choosing to use donor eggs instead of IVG are explored and assessed. It is concluded that egg donation would only be ethically justified in a narrow range of special cases given the (hypothetical) availability of IVG treatment and, further, that egg donation could itself be replaced by donation through IVG techniques. Two possible criticisms of this position are then considered: Ones based on respect for patient wishes, and on loss of donor benefit. It is concluded that whilst neither argument constitutes a strong enough reason to continue with programmes of egg donation, egg‐sharing programmes could still be permitted come the advent of IVG; these could then provide a morally acceptable source of “natural” donor eggs.

## 1 INTRODUCTION AND BACKGROUND

Discussions in the broader literature regarding the emerging biotechnology of in vitro gametogenesis (IVG)^1^ suggest high hopes for the possibilities of the intervention; from the use of the process to facilitate access to embryos for research purposes,^2^ to enabling people who currently rely on gamete donation to reproduce to have genetically related children.^3^ As Smajdor writes: “The successful development of [artificial gametes] for reproductive purposes would mean that gametes could be obtained using genetic material from the prospective parents, whether or not they ever had viable gametes of their own.”^4^ That people who currently rely on the use of donor gametes to reproduce would be able to have genetically related children with IVG is an exciting prospect, and one which in turn raises questions regarding the future of gamete donation. This paper explores these with a focus on egg donation in particular, and asks: If IVG were safe, effective and inexpensive, when (if ever) would egg donation be ethically justifiable?

One might be inclined to wonder at this point whether there could indeed be instances where a fertility patient would still prefer to use donated eggs, even where IVG was available and cost‐effective, and had been shown to be safe. This is not unthinkable, and it is an important question to ask. There is of course a practical aspect to this, for if there were no demand for egg donation then both public and private healthcare organizations would need to respond accordingly. But this possibility raises ethical questions as well, as indicated by the question above—the answer to which would of course inform whether it would be ethically acceptable to continue to pursue egg donation once IVG has reached a point where it is safe, effective and cost‐effective. Further, the question and corresponding discussion also provide a new context in which broader questions around both IVG and egg donation can be explored. The assumption in the literature seems to be that IVG will eliminate the need for gamete donation if it is found to be safe, effective, inexpensive and ethically acceptable. As such, there has been no consideration of whether there might in fact be demand for gamete donation to continue in parallel to provision of IVG, or to what the ethical implications of this would be in the context of egg donation in particular (given the invasiveness of the process and the risks and harms involved). Some of the reasons why people might prefer donated eggs to the use of IVG are explored in the broader literature—in particular the view that the latter intervention is “unnatural,” and concerns regarding the psychosocial impact that being born through IVG might have on the resulting child. These are provided as possible criticisms of IVG more broadly, and while this will be true of their use here, the context in which they are being discussed in this paper (the ethical acceptability of providing egg donation as an *alternative* treatment option) is different to how they have been previously approached. This paper explores issues and concerns that might inform a recipient’s reasoning in choosing to use donor eggs instead of eggs produced through IVG (even though the latter would lead to the creation of children to which the recipient would be genetically related). This section concludes with the assertion that egg donation can only be ethically justified in specific, serious cases given the (hypothetical) availability of IVG treatment, and further, that even then egg donation as we know it today could be replaced by donation through IVG techniques. Following this, two possible replies to this conclusion—respect for patient wishes, and the loss of donor benefit—are explored.

This paper therefore considers some issues that are relevant today—the harms and risks involved in egg donation—and explores them within the context of the emerging field of IVG. Currently, if the intended mother is unable to produce her own eggs then there is no alternative method for her to have her own genetically related children^5^—in short, if eggs are needed then egg donation is the only way to acquire them.^6^ However, IVG could offer an alternative: Allowing people who cannot provide their own eggs to have eggs created from their stem cells and to have a genetically related child. This paper in turn forms part of a broader discussion on the replacement of current biomedical interventions with emerging technologies, particularly the use of “artificial” forms of organic articles such as organs and organoids^7^ (which could eliminate the need for human organ donors and aid pharmaceutical research), and of course gametes.

### 1.1 IVG and egg donation

IVG refers to the use of stem cell technology to create eggs and sperm. There has been some success in trials with mice^8^ and there is hope that IVG will one day be viable for human use, both for research and reproduction. This said, IVG is not yet available, so discussions on the topic are necessarily speculative in nature. Indeed, for simplicity’s sake, this paper adopts a hypothetical point of departure wherein it is assumed that IVG is (or rather will be) safe, effective and cost‐effective. This approach will serve to facilitate discussion by putting to one side topics already explored in the literature^9^ in favour of exploring the issues and questions raised here. Further, this paper is not concerned with the moral status of IVG itself, but rather with whether egg donation can be ethically justified when a sufficiently good alternative (IVG) is available. Cases in which a woman undergoes the medical procedures related to egg harvesting with the sole intention of going on to donate those eggs will be the principal focus of this work; other kinds of egg donation, such as egg sharing and the donation of eggs leftover following reproductive treatment, will be addressed later in the paper. The decision to focus on egg donation in particular (as opposed to gamete donation more broadly) is due to the harms, risks and greater effort from donors that are involved in egg donation, in contrast to the risk‐free and simple process of sperm donation. These considerations therefore make the discussions surrounding this type of reproductive donation more urgent; for whilst sperm donation involves an extremely simple process, egg donation is necessarily a far more medicalized procedure.^10^ The procedure is also one that is not without risks. The London Egg Bank notes some of these: Pelvic infection, bowel, bladder or vessel perforation, ovarian hyperstimulation syndrome, and adverse reactions to medication (including concerns over a possible (though unconfirmed) risk of eventual development of related cancers).^11^ Many studies indicate that the risks are low, with one study putting the rate of “serious complications” (including “ovarian hyperstimulation syndrome, ovarian torsion, infection, and ruptured ovarian cyst”^12^) following donation as being as low as 0.7%.^13^ The same study puts the rate of minor complications serious enough to move the donor to seek medical attention at 8.5%.^14^ This demonstrates that whilst the risks of encountering complications as a result of undergoing egg donation are quite low, they are nonetheless very real and can be serious. It is also important to note the more general harms involved in egg donation, be it the inconvenience involved in preparing for donation (such as daily hormone injections, regular trips to the fertility clinic for ultrasonography and blood tests,^15^ and a requirement that donor abstains from sex during the process^16^), or indeed the invasive procedure used to retrieve the eggs.^17^

## 2 THE CASE AGAINST EGG DONATION

This section argues against the continuation of egg donation once (safe, effective and inexpensive) IVG becomes a feasible alternative. This is achieved by exploring reasons why would‐be recipients might opt for the use of donor eggs over having IVG (even at the expense of genetic relatedness), before arguing that the majority of these are not sufficient to justify egg donation.

### 2.1 “Naturalness”

One reason why people might decide to opt to use donor eggs as opposed to IVG is to avoid that which they perceive to be “unnatural”; for as Testa and Harris note, some people view the “natural” as being “superior to the artificial or synthetic.”^18^ Also, some people might reject IVG treatment in favour of “the natural” alternative of donor eggs, due to having a so‐called “yuck” response to the idea of IVG (or of using stem cells more broadly^19^), and so might therefore be inclined towards the use of donated eggs, even where this would mean losing the genetic link to their future child. That this view could be held by some recipients seems to be confirmed by Hendriks and colleagues (although their study was about donor sperm rather than eggs); their findings indicate that, “The more importance couples attached to naturalness and moral acceptability, the less likely they were to opt for [artificial gametes].”^20^ Further, of patients asked whether they would consider IVG treatment (if available and safe) as a “first and/or last resort treatment option,” a minority said they would not, with 46% of that group preferring alternative treatment such as the use of donor sperm instead.^21^

However, as many writers point out, “natural” does not always mean “better”—practically or morally.^22^ As Testa and Harris note: “the natural *per se* is often very harmful; disease, mutations, pestilence, floods, hurricanes, fire, landslides and the like can cause massive loss of human life and do terrible damage to the environment.”^23^ They argue as well that much of medicine could be considered to be “unnatural,” citing the example of the heart drug digoxin, which (whilst having a natural foxglove base) is “always given in a highly purified pharmaceutical form.”^24^ Therefore, the argument can be made that unless one is willing to consider a “natural” disease to be morally preferable to the “unnatural” medicines that treat it, it must be agreed that the natural is not necessarily practically or morally better than the unnatural. This is not to say that the *un*natural is practically or morally better than the natural, but rather that there is *no intrinsic moral difference between the two*. As John Stuart Mill writes: “The scheme of Nature, regarded in its whole extent, cannot have had, for its sole or even principal object, the good of human or other sentient beings. What good it brings to them is mostly the result of their own exertions.”^25^

Furthermore, it is important to note that “the unnatural” is ubiquitous in the world today; indeed, even farm produce cannot be considered completely natural, given the selective breeding, cloning and even induced mutation that has occurred over the centuries.^26^ And just as the use of IVG techniques in reproduction might be considered “unnatural,” so too could the use of a donated egg—as its use would similarly involve interventions which arguably are not “natural”:^27^ The egg retrieval procedure itself (and the preparation leading up to the procedure), the IVF techniques used to produce an embryo with the donated egg, and the transfer of the embryo into the uterus of the gestational mother.

This said, one might argue that there exists a sliding scale of “naturalness”—that something that is perceived as “unnatural” could nevertheless be seen as *more natural* (or *less unnatural*) than another “unnatural” intervention. So then a critic might argue that egg harvesting and IVF may well be unnatural but less so than IVG interventions and so should be preferred. However, as already noted, the natural could at best be said to be morally neutral and so the notion that one intervention might be considered to be more natural (or less unnatural) than another is not morally relevant in any case.

So then the “naturalness” argument fails both due to its faulty premise (that the natural is morally better to the unnatural), and because it is incoherent. That IVG can be said to be “unnatural” is not a reason for moral objection to the endeavour, and it is certainly not a reason to favour egg donation, especially given that it too involves “unnatural” processes. As Testa and Harris write: Natural reproduction may be more enjoyable than synthetic reproduction, or cheaper, or safer or more private, these are reasons to prefer it. But there is no reason to prefer a natural process simply because it is natural.^28^

### 2.2 Parental preference for specific traits

Another reason for preferring donor eggs arises in cases where people want their child to inherit certain beneficial genetic traits from a gamete donor. Parallels could be drawn here with the use of the so‐called “genius sperm bank” (the Repository for Germinal Choice)^29^ where women were given the opportunity to undergo donor insemination with the sperm of gifted men; an offer taken up not only by single women, but also by couples.^30^ This same reasoning could underlie a decision to use donated eggs, especially where the donor in question possesses “desirable” traits (such as intelligence or athleticism), as opposed to preferring one’s own genetic material and so using IVG.

However, the “genius sperm bank” example above is relevantly different from egg donation, because egg donation is far more harmful, risky and medicalized than sperm donation. Furthermore, Bourne and colleagues^31^ argue that IVG technology would in fact aid this pursuit for specific traits as it would allow scientists to make huge numbers of embryos from which desired traits could be identified and corresponding embryos chosen for transfer, without the need to use donor eggs.^32^ As such, wanting desirable traits does not ethically justify egg donation, especially given the harms and risks of the procedure.

### 2.3 Avoiding negative psychosocial impact

Some people might opt for donor eggs rather than IVG because of concerns about the psychosocial impact that being the product of IVG interventions might have on the resulting child.^33^ There may be worries, for instance, that the child in question would be more disturbed by the knowledge that they are the result of (so‐called) “artificial gametes” than by the knowledge that they are the child of an egg donor (and so their social mother is not their genetic mother). There could also be concerns regarding the reactions of others to the resulting child, especially if there is a “yuck” reaction to IVG in the general public.

One possible way of dealing with this concern is offered by Mertes and Pennings, who suggest that parents could simply refrain from telling their children that they were the product of IVG if they were concerned about the impact that the knowledge could have—especially as, unlike in the case of egg donation, both social parents would pass any genetic paternity or maternity test. Making the comparison with IVF, they write: “There is a general consensus that there are no strong reasons why a child should be informed that it is the result of in vitro fertilization.”^34^ However, this is problematic, as there would of course be a risk that the child might one day discover her origins and feel betrayed by her parents’ dishonesty. Further, the child’s own sense of identity might in turn be affected by the revelation, especially if she or those in her community took a dim view of the intervention. Writing with reference to mitochondrial replacement therapy (MRT), Scully argues that even if the child never did find out the truth, she could still be affected through her relationships with those that *did* know of her origins: “problems might also arise even if the child does *not* know how she came to be born, if the parents’ or family’s knowledge that MRT were used changes how they relate to her.”^35^ One could argue as well that secrecy could indirectly contribute to stigma around the use of IVG for reproductive purposes, as openness can help normalize the intervention, both in close social groups (such as families)^36^ and in the wider community. Such secrecy, however well intentioned, could therefore impede this normalization of the intervention.

It is this normalization that will be key, and education will play a crucial role in this endeavour, be that education in an institutional sense (for instance, schools and professional training) or through popular media. Referring to studies into public perceptions of medical interventions, Gilbert and colleagues note that: “mass media influences patient education, comprehension, and understanding of health issues. In that respect, content and stereotypes disseminated through mass media influence prospective patients’ hopes and expectations.”^37^ The representation of IVG in the media will then clearly impact on the public perception of the intervention. With regards to public perceptions of *people* born through IVG techniques, whilst perceptions of the intervention itself will likely play a role here, media representation will again be an important factor both in informing opinion and in normalizing the use of the technology and the existence of people born from it. As Scully (again with reference to MRT) writes:

If identity is created in part through cultural narratives, and can be damaged in a morally significant way by the lack of a ‘good story’, then ensuring that such good stories are available to MRT children and their families becomes a collective moral responsibility... it may become necessary to establish a form of systematic monitoring to follow how the media and other social institutions discuss MRT children and families, and engage with these agencies to counter potentially hurtful, harmful or limiting identity stories with more nuanced ones based on accurate empirical knowledge.^38^

Language will also be important; talk of “artificial” or “synthetic” gametes is not only inaccurate^39^ but could also prove harmful, encouraging the misconception that those born of these “artificial” gametes are therefore “artificial” people. Concerns around the misleading nature of terms used in the media were also raised with regards to MRT.^40^

So whilst concerns regarding the psychosocial impact of being born of IVG are reasonable, they may well be assuaged through public education—especially by way of the media and through the avoidance of certain provocative (and indeed misleading) terms such as “artificial” or “synthetic” gametes. Through these methods, public^41^ perception of IVG and of those born as a result of the intervention can be altered and the endeavour normalized. If successful, the psychosocial impact may well be so minor that it would not then constitute grounds to justify the use of donor eggs over IVG.^42^

### 2.4 Avoiding disease

Concerns regarding heritable illnesses or conditions might inform a recipient’s decision to opt for using donor eggs as opposed to having IVG—for instance, where the intended parent has a predisposition to some hereditary condition. This would constitute an ethically justifiable reason for pursuing egg donation. It could be countered that preimplantation genetic diagnosis (PGD) should be pursued first in order to try to identify affected embryos and prevent their transfer; however, one might reply that PGD is imperfect and might not successfully identify all conditions all of the time. However, further research into PGD^43^ may well improve the technique to the extent that hereditary illnesses or conditions no longer pose a concern in such instances, but where PGD would be ineffective (or unreliable) then the use of donated eggs would be considered ethically justified.^44^

Related to this, there may be cases where a recipient would opt to use donor eggs where there is a risk of mitochondrial disorder in resulting offspring. As Smajdor and Cutas note: “[w]omen with mitochondrial mutations would still need to derive their mitochondrial DNA from other sources.”^45^ As a result, a donor egg would be required in order to utilize the mitochondrial transfer technology needed to protect against mitochondrial disease in resulting children. This is another case where the use of donor eggs would be ethically acceptable and justified; the good achieved from avoiding the harm of mitochondrial diseases could easily be seen as justification for the (comparatively far lesser) harms involved in egg donation.

### 2.5 Conclusion to Section 2

Given the cases listed above, it seems that the only instances of egg donation which could be morally justified are those where mitochondrial transfer would be required, or where there are concerns regarding heritable illnesses or conditions that cannot be dealt with using PGD (or indeed where there is a moral objection to the methods involved in PGD). This being said, perhaps egg donation (as we know it today) could be replaced with IVG techniques. So if a donated egg was required (for instance, for use in mitochondrial transfer) then the would‐be egg donor could instead donate skin cells (for instance) from which eggs could be derived, as opposed to undergoing the invasive interventions currently used in egg donation and the related harms and risks.

Of course it could be the case that the recipient of the donated egg (created through IVG techniques) would have concerns about “naturalness”, or of what to tell the resulting children with regards to their origins. However, as stated above, such concerns are not sufficient to justify the use of egg donation (as it is today) in any other circumstances, and so we might be unwilling to make an exception here as well. Although, as I will discuss in the following section, there may yet be alternative options available for those who would still prefer the use of “natural” donor eggs.

## 3 RESPONSES IN DEFENCE OF EGG DONATION

This section considers two potential criticisms of the conclusions drawn in the previous section. The first suggests that we should offer the alternative of donor eggs to recipients as a way of *respecting their wishes* in this matter (drawing parallels with the use of bloodless surgery in treating Jehovah’s Witnesses). The second moves the focus from would‐be egg recipients to egg *donors*, and considers the argument that the donors themselves would be disadvantaged should IVG mark the end of egg donation as we know it today.

### 3.1 Respect for wishes

One could argue that it would be wrong to rule out the use of egg donation as we know it today as we should strive to accommodate and respect the wishes of patients (and would‐be recipients) where possible; parallels can be drawn with Jehovah’s Witnesses here. The Jehovah’s Witnesses’ interpretation of the Bible leads them to forbid their members to receive blood transfusions, so members often refuse the intervention. As a result, some surgeons endeavour to use specialist “bloodless surgery” techniques in order to be able to treat these people whilst still respecting their views and wishes.^46^ One might then argue that this approach of “going the extra mile” in order to respect the wishes and beliefs of a patient in their treatment should be applied to those receiving fertility treatment who object to the use of gametes produced through IVG techniques. In short, one could ask whether we should respect the wish to use donor eggs rather than IVG just as we do the request to have bloodless surgery: To try to accede to it, even where it is costly.

This parity argument has some grounding in the broader literature; for instance, Juth and Lynøe write that there are no morally relevant differences between bloodless surgery and the uncommon intervention of hymen restoration,^47^ and so argue that there should be parity in the provision of the two procedures.^48^ Does the same argument apply to provision of “natural” donor eggs? No—the cases are not morally equivalent, for two main reasons.

First, in the case of bloodless surgery, this involves costs such as financial costs, time costs and pressure on resources; however, whilst the use of donated eggs involves these costs as well, it also involves the further cost of direct harms to an individual: The donor.^49^ This then suggests that bloodless surgery is disanalogous to the use of donor eggs (especially where an alternative such as IVG is available).

Second, a further difference between these cases is that of the risk of societal isolation and cultural shaming—for if a Jehovah’s Witness accepts a blood transfusion, in addition to religious concerns about what this might mean for the patient’s soul, there is a risk that the individual in question would be shunned and cast out from their societal group (which can also involve separation from family members). It could be argued that this gives further weight to the patient’s decision to opt for bloodless surgery. When discussing cases where the recipient desires to use non‐IVG donor eggs, she is unlikely to face the same consequences. This said, it is unclear how some social and indeed religious groups might react to IVG technology when it comes into common use; it could well be that some religions ban use of the technology by its members under penalty of shunning or shaming—much like Jehovah’s Witnesses and blood transfusions. If this does become the case then there would be a far stronger argument for parity on these grounds.

It is also interesting to note that surgeons can choose not to treat patients who refuse blood transfusions (if it is not an emergency). As the Royal College of Surgeons explains: “Surgeons have the right to choose not to treat patients if they feel that the restrictions placed on them by the refusal of blood products are contrary to their values as a doctor.”^50^ It may be the case that some doctors would refuse to treat fertility patients who opt for donor eggs because of a preference for the natural, if they feel that acceding to such a request would be “contrary to their values as a doctor,” especially given the reasonable alternative available (IVG). This could be the case if, for instance, the doctor in question felt concern regarding the welfare of egg donors.^51^ This also indicates another difference between the two cases: There can be *emergencies* in which bloodless surgeries are required (and the physician’s conscientious objection negated), but the same cannot be said of the preference for donated eggs, use of which would constitute only a quality‐of‐life intervention, not a life‐or‐death one.

In any case, fertility patients would have their refusal to use IVG‐produced eggs respected, as “[a]ll patients in the UK with mental capacity have the absolute legal and ethical right to refuse treatment or any aspect of treatment.”^52^ But this need not mean that patients would then have an automatic right to then access “natural” donor eggs, nor would respecting wishes form strong enough grounds to justify egg donation more generally.^53^

### 3.2 Donor benefit

A second criticism is that egg *donors* would be disadvantaged if IVG were to spell the end of egg donation, since they themselves can be said to benefit from donating: Be that financially (by way of payment or through egg‐sharing^54^ schemes), or in terms of the more intangible benefits gained from egg donation.^55^

First, with regards to the financial benefits gained from egg donation, it is important to note that the financial compensation of egg donors is a controversial endeavour, with the ethical issues and concerns raised by the practice being well explored in the literature.^56^ Further, would‐be egg donors could instead be financially compensated for donations of genetic material (such as skin cells) for use in reproductive IVG and so would not necessarily lose this benefit. That said, the amount offered to egg donors as financial compensation can be quite high^57^ in acknowledgement of the invasive and risky nature of the procedure—which would be absent in the case of IVG donation, where the procedure would be less invasive and burdensome than even blood donation. As such, the high payments sometimes associated with egg donation might seem too generous once the procedure has moved from the methods used today to those of IVG.

Further, market forces could come into play. There is currently a shortage of donated eggs in the UK^58^ which will inform the financial value attached to each cycle; however, IVG technology could change this state of affairs quite rapidly, as it would enable scientists to produce vast numbers of eggs. Therefore, in stark contrast to the situation today, we would essentially have a flooded market of eggs, which would in turn affect the financial value attached to egg donation, meaning that the large monetary amounts sometimes offered would no long be available in any case. Finally, the use of IVG techniques would mean that egg donation would become less invasive and labour‐intensive than sperm donation^59^—and so those who donate eggs through IVG^60^ could perhaps have grounds to lay claim only to a similar amount of compensation to that which is offered to sperm donors, or perhaps even less.

Another financial benefit of which donors might be deprived is the option to participate in egg sharing schemes. As Gürtin and colleagues explain: “Egg sharing is the name given to the scheme whereby an IVF patient gives a portion of her eggs to an anonymously matched recipient in exchange for subsidised or free fertility treatment.”^61^ However, such programmes are not without controversy, especially in cases where egg donors would be otherwise unable to afford fertility treatment, as this then raises questions regarding undue inducement and even exploitation (although these concerns are already attended to in the literature^62^). Should the advent of IVG spell the end of egg donation (as we know it) then one could be concerned that this would also mean the end of egg‐sharing schemes, clearly affecting those women who cannot afford fertility treatment without such programmes in place. But would this be the case?

First, it is important to note that much will depend on how IVF would proceed in women who do not require donor eggs once IVG technology has become available. That is, whether these women would have their eggs retrieved through methods currently used today, or whether they would be provided with IVG treatment regardless (so as to eliminate the harms and risks associated with egg retrieval); in either case the woman in question could be offered the choice of donating excess eggs to offset the cost of her IVF treatment (as is currently done).^63^ If the donor has chosen to have her eggs retrieved using current methods, this would then allow those persons who cannot produce their own eggs, and who have a strong preference for non‐IVG eggs, to obtain such gametes from these egg‐sharing donors.

With regards to the more intangible benefits of donation, there are two responses. First, egg donation even as it is today might not be entirely unjustifiable. As I concede above, egg‐sharing schemes could be justified even where IVG interventions are available. Also, known donor donation could possibly be considered justifiable as well (although less so, as the harms involved would still remain)—for if the egg donor in question knew the reasons behind the donation (even if those reasons have been deemed here as insufficient to justify the intervention) but was still willing to donate for these reasons, then that would be her own fully informed decision.^64^ Also, again, the need for donation might not be eradicated entirely as there may still be call for donated genetic material (such as skin cells) from which gametes can be created through IVG. Therefore, donors could still obtain these benefits from donation, including those that are intangible, from this far less invasive procedure.^65^ A parallel could be drawn here with blood donation—a relatively simple and non‐invasive procedure for which people are not financially compensated in many countries.^66^

## 4 CONCLUSION

This paper forms part of a broader ongoing debate about the replacement of current biomedical interventions with emerging technologies—in particular the use of stem‐cell‐derived forms of organic objects such as organs and gametes. It explores whether, and under what circumstances, egg donation would be ethically justifiable should IVG technology for reproductive purposes become inexpensive, effective and safe. Section 2 concludes that avoiding risk of disease provides the only reasonable justification for using donor eggs and, even then, IVG‐based egg donation should be preferred. Section 3 adds the caveat that non‐IVG egg donation could also be ethically justifiable when the eggs have been obtained through egg‐sharing schemes; this is because the donor in such cases would have undergone the risks and harms involved in egg retrieval in any case for her own medical benefit. This also applies to other cases where the donor has not undergone any further risks and harms; for example, the donation of eggs left over following reproductive treatment.

## CONFLICT OF INTEREST

The author declares no conflict of interest.

## Footnotes

1 I explain this in more detail below, but, in short, IVG involves the use of stem cell technology to create gametes.

2 Suter, S. M. (2015). In vitro gametogenesis: Just another way to have a baby? *Journal of Law & the Biosciences*, *3*, 87–119.

3 Ibid. There are other, more controversial possible uses that have been previously explored in the literature, including ‘multiplex parenting’ (where more than two parents are genetically related to the same child—see Palacios‐González, C., Harris, J., & Testa, G. (2014). Multiplex parenting: IVG and the generations to come*. Journal of Medical Ethics*, *40*, 752–758) and ‘solo IVG’ where one person is the sole genetic parent of a child (see Cutas, D., & Smajdor, A. (2016). ‘I am your mother and your father!’ In vitro derived gametes and the ethics of solo reproduction. *Health Care Analysis*. doi:10.1007/s10728‐016‐0321‐7). The latter of these could have relevance to discussions in this paper, however I have decided to put it to one side for simplicity’s sake (especially as there is no guarantee that ‘solo IVG’ would be legally permitted in the future in any case).

4 Smajdor, A., & Cutas, D. (2015). Will artificial gametes end infertility? *Health Care Analysis*, *23*, 134–147: p. 135. It is prudent to note that IVG‐generated gametes are sometimes referred to in the literature as ‘synthetic’ or ‘artificial’ gametes; for the most part, these terms have fallen out of favour in more recent years given their misleading nature and also negative connotations.

5 If a donor egg is used then the mother is not genetically related to the child (with the possible exception of where a donor has been used in order to pursue mitochondrial transfer interventions), however she may gestate the pregnancy as well as act as social mother after the birth.

6 Either from a friend or relative who volunteers to donate their eggs (a ‘known donor’), from egg‐sharing schemes (as I explore in Section 3), or from egg donation in general.

7 See, for instance, Vermeulen, N., Haddow, G., Seymour, T., Faulkner‐Jones, A., & Shu, W. (2017). 3D bioprint me: A socioethical view of bioprinting human organs and tissue. *Journal of Medical Ethics*. doi:10.1136/medethics‐2015‐103347.

8 Suter, op. cit. note 2.

9 For an overview of topics explored and the broader literature on IVG, see Smajdor, A., & Cutas, D. (2015). *Artificial gametes. Nuffield council on bioethics: Background paper*. London. Retrieved July 10, 2017, from http://nuffieldbioethics.org/wp-content/uploads/Background-paper-2016-Artificial-gametes.pdf

10 The following sources provide some detail regarding the onerous process involved in egg donation: London Egg Bank. (2013). *The London Egg Bank: All you need to know*. London. Retrieved August 19, 2017, from http://www.londoneggbank.com/images/uploads/London_Egg_Bank_Brochure.pdf; also Sauer, M. (2006). Oocyte and embryo donation 2006: Reviewing two decades of innovation and controversy. *Reproductive Biomedicine Online*, *12*, 153–162; London Egg Bank; *How do I become an egg donor?: Step‐by‐step process*. London. Retrieved August 10, 2017 from http://www.londoneggbank.com/how-do-i-become-an-egg-donor/step-by-step-process/

11 London Egg Bank, op. cit. note 10.

12 Maxwell, K. N., Cholst, I. N., & Rosenwaks, Z. (2008). The incidence of both serious and minor complications in young women undergoing oocyte donation. *Fertility & Sterility*, *90*, 2165–2171: p. 2165.

13 Ibid.

14 Ibid.

15 London Egg Bank, op. cit. note 10.

16 Sauer, op. cit. note 10.

17 London Egg Bank, op. cit. note 10.

18 Testa, G., & Harris, J. (2005). Ethics and synthetic gametes. *Bioethics*, *19*, 146–166: p. 161.

19 However, this could well be due to the use of foetuses/embryos in stem cell research, rather than the stem cells themselves; that being said, the stigma may well carry through.

20 Hendriks, S., Hessel, M., Mochtar, M. H., Meissner, A., van der Veen, F., Repping, S., & Dancet, E. A. F. (2016). Couples with non‐obstructive azoospermia are interested in future treatments with artificial gametes. *Human Reproduction*, *31*, 1738–1748: p. 1742.

21 Ibid.

22 See for instance Testa & Harris, op. cit. note 20; also A. Smajdor & D. Cutas. Artificial Gametes: The Unnatural and the Artefactual. *J Med Ethics* 2018; doi: 10.1136/medethics‐2017‐104351. This is epecially the case given that the intervention in question (IVG) is assumed here to be safe and so neither mother nor child would be in danger.

23 Testa & Harris, op. cit. note 18, p. 162.

24 Ibid.

25 Mill, J. S. (2009). *Three essays on religion* (p. 103). Peterborough: Broadview Press.

26 Wieczorek, A. M., & Wright, M. G. (2012). *History of agricultural biotechnology: How crop development has evolved*. Cambridge, MA: Nature Education. Retrieved May 2, 2018, from https://www.nature.com/scitable/knowledge/library/history-of-agricultural-biotechnology-how-crop-development-25885295

27 Paxson, H. (2003). With or against nature? IVF, gender and reproductive agency in Athens, Greece. *Social Science & Medicine*, *56*, 1853–1866.

28 Testa & Harris, op. cit. note 18, p. 161.

29 Also referred to as the ‘Nobel Prize Sperm Bank’. See Morrice, P. (2005, 1 July). *The genius factory: The curious history of the Nobel Prize Sperm Bank*. New York Times. Retrieved May 1, 2018, from https://www.nytimes.com/2005/07/01/arts/the-genius-factory-the-curious-history-of-the-nobel-prize-sperm-bank.html

30 Olding, P. (2006, 15 June). *The genius sperm bank. BBC news*. London. Retrieved June 6, 2017, from http://news.bbc.co.uk/1/hi/magazine/5078800.stm. However, it is not clear whether any of these couples would have been able to conceive genetically related children and so were using the sperm bank as an alternative choice (as would be the case in recipients offered IVG but choosing to opt for donor eggs); as such, the comparison here might be imperfect.

31 Bourne, H., Douglas, T., & Savulescu, J. (2012). Procreative beneficence and in vitro gametogenesis. *Monash Bioethics Review*, *30*, 29–48.

32 However, they note as well that there could of course be concerns raised here with regards to the moral status of the embryo (or even gametes used), especially as this process would involve discarding unsuitable embryos.

33 Many but especially Mertes, H., & Pennings, G. (2010). Ethical aspects of the use of stem cell derived gametes for reproduction. *Health Care Analysis*, *18*, 267–278.

34 Ibid: 275.

35 Scully, J. L. (2017). A mitochondrial story: Mitochondrial replacement, identity and narrative. *Bioethics*, *31*, 37–45: p. 40.

36 Ibid.

37 Gilbert, F., Viaña, J. N. M., O’Connell, C. D., & Dodds, S. (2018). Enthusiastic portrayal of 3D bioprinting in the media: Ethical side effects. *Bioethics*, *32*(2), 94–102.

38 Scully, op. cit. note 35, p. 45.

39 Smajdor & Cutas, op. cit. note 9.

40 Harris. J. (2012, 19 September). Misleading talk of 'three‐parent babies' helps no one. *The Guardian*. London. Retrieved December 16, 2017, from https://www.theguardian.com/commentisfree/2012/sep/19/misleading-three-parent-babies-gene-therapy

41 Or at least the majority of members of the public.

42 This being said, I will concede that there will be some people or communities where these efforts may well be in vain, and where this would directly affect an individual (for instance, where they are at risk of being disowned or shunned—see Section 3) then this would form more reasonable grounds for considering the use of donor eggs. However, for the most part, I still assert that education and positive media representation will resolve many of the concerns regarding psychosocial harm.

43 Which may be facilitated by the advent of IVG technology (as the availability of embryos for experimentation would increase)—see Suter, op. cit. note 2.

44 Indeed this could also be said in the cases of those persons who have strong concerns with regards to the disposal aspect of PGD.

45 Smajdor & Cutas, op. cit. note 4, p. 138.

46 Schaffer, A. (2015, 12 August). How Jehovah’s Witnesses are changing medicine*. The New Yorker*. New York, NY. Retrieved July 10, 2017, from http://www.newyorker.com/news/news-desk/how-jehovahs-witnesses-are-changing-medicine

47 Which, as they note, is sometimes requested by women in fear of cultural shaming (or even of ‘honour’‐related violence).

48 Juth, N., & Lynøe, N. (2014). Are there morally relevant differences between hymen restoration and bloodless treatment for Jehovah’s Witnesses? *BMC Medical Ethics,* *15*, 89. doi:10.1186/1472‐6939‐15‐89

49 Though one could argue that financial, time and resource costs are still harms in a broader sense, as more money, time and resources spent on one patient means that there is less money, time and resources available for other patients.

50 Royal College of Surgeons. (2016). *Caring for patients who refuse blood: A guide to good practice for the surgical management of Jehovah’s Witnesses and other patients who decline transfusion* (p. 8). London: Royal College of Surgeons. Retrieved September 10, 2017, from https://www.rcseng.ac.uk/library-and-publications/college-publications/docs/caring-for-patients-who-refuse-blood

51 This may of course raise questions regarding the duties of doctors to persons who are not their patients, which would be beyond the scope of this paper.

52 Ibid: 9.

53 Although, if there would indeed be risk of religious or social exclusion, then this could constitute a stronger justification.

54 Where women undergoing IVF with their own eggs are offered the option to donate excess eggs to other fertility patients in exchange for subsidised treatment.

55 Such as, for example, the positive feelings that may come from the knowledge that the donation has helped someone to conceive a much‐wanted child.

56 See, for example, Kenney, N. J., & McGowan, M. L. (2010). Looking back: Egg donors’ retrospective evaluations of their motivations, expectations, and experiences during their first donation cycle. *Fertility & Sterility*, *93*, 455–466; also, Daniels, K. R. (2000). To give or sell human gametes—The interplay between pragmatics, policy and ethics. *Journal of Medical Ethics*, *26*, 206–211; Shenfield, F., & Steele, S. J. (1995). A gift is a gift is a gift, or why gamete donors should not be paid. *Human Reproduction*, *10*(2), 253–255; The Ethics Committee of the American Society for Reproductive Medicine. (2007). Financial compensation of oocyte donors. *Fertility & Sterility*, *88*, 305–309.

57 For example, U.K. donors are offered £750 per cycle (see Human Fertilisation and Embryology Authority. *Donating Your Eggs*. London. Retrieved November 14, 2017, from https://www.hfea.gov.uk/donation/donors/donating-your-eggs/), while in the United States, although the amount can vary widely, some companies advertise payments of as much as $10,000 per cycle (Egg Donor America. (2018, 27 April). Egg donor compensation. Annandale, VA. Retrieved May 1, 2018 from https://www.eggdonoramerica.com/become-egg-donor/egg-donor-compensation.

58 Hepburn, J. (2017, 3 May). Behind the latest IVF furore lies untold human suffering*. The Guardian*. London. Retrieved June 27, 2017, from https://www.theguardian.com/commentisfree/2017/may/03/fertility-treatment-ivf-selling-eggs

59 Which itself only attracts financial compensation of up to £35 per clinic visit (plus expenses) in the U.K.: Jessop Fertility. (2017). *Sperm Donation Information*. Sheffield. Retrieved August 8, 2017 from http://www.jessopfertility.org.uk/donor/sperm-donation-information#do-i-get-paid

60 That is, skin cells (for example) from which eggs can be created.

61 Gürtin, Z. B., Ahuja, K. K., & Golombok, S. (2012). Egg sharing, consent and exploitation: Examining donors’ and recipients’ circumstances and retrospective reflections. *Reproductive Biomedicine Online*, *24*, 698–708: p. 698.

62 See for instance Ibid, and Haimes, E., Taylor, K., & Turkmendag, I. (2012). Eggs, ethics and exploitation? Investigating women’s experiences of an egg sharing scheme. *Sociology of Health & Illness*, *34*, 1199–1214.

63 Excess eggs generated from IVG techniques could also be donated, for instance to fertility patients who require a donor egg but do not mind that it was made through these means.

64 Although the use of known donors carries its own issues with regards to concerns of coercion, which will need to be dealt with on a case‐by‐case basis (see Sauer, op. cit. note 10). This also raises questions relating to donor autonomy more broadly. Unfortunately, I lack the space to provide an in‐depth exploration of this topic—though it is well attended to in the broader literature.

65 If someone is inclined at this juncture to deny that they would get the same sense of ‘doing good’ or of having helped others from donating skin cells as they would from donating eggs then this would surely raise other questions, such as whether the labour involved in such donation is morally relevant, and perhaps even what motives are in play in the donor’s decision‐making. Unfortunately, space forbids a more in‐depth exploration of these questions.

66 The World Health Organization (WHO) states that ‘74 countries collect over 90% of their blood supply from voluntary unpaid blood donors’ and further that this figure ‘includes 57 countries with 100% (or more than 99%) of their blood supply from voluntary unpaid blood donors.’ See: WHO. (2017). *Blood Safety and Availability: Fact Sheet*. Geneva, Switzerland: WHO. Retrieved June 20, 2017, from http://www.who.int/mediacentre/factsheets/fs279/en

